# Salvage brachytherapy in prostate local recurrence after radiation therapy: predicting factors for control and toxicity

**DOI:** 10.1186/1748-717X-9-102

**Published:** 2014-04-30

**Authors:** Iván Henríquez, Gemma Sancho, Asunción Hervás, Benjamin Guix, Joan Pera, Cristina Gutierrez, Oscar Abuchaibe, Rafael Martínez-Monge, Alejandro Tormo, Alfredo Polo

**Affiliations:** 1 Department of Radiation Oncology, University Hospital of Sant Joan. Institute d’Investigació Sanitaria Pere Virgili (IISPV), Reus, Tarragona, Spain; 2 Department of Radiation Oncology, Hospital de la Santa Creu i Sant Pau, Barcelona, Spain; 3 Department of Radiation Oncology, Brachytherapy Unit, Hospital Ramón y Cajal, Madrid, Spain; 4 Department of Radiation Oncology, Radiation Oncology Medical Institute, IMOR, Barcelona, Spain; 5 Department of Radiation Oncology, Brachytherapy Unit, Institut Catalâ d’Oncologia, Barcelona, Spain; 6 Department of Radiation Oncology, Virgilio Galvis Ramirez Cancer Centre, Bucaramanga, Colombia; 7 Department of Radiation Oncology, University Clinic of Navarra, CUN, Pamplona, Spain; 8 Department of Radiation Oncology, University Hospital Politècnic La Fe, Valencia, Spain

**Keywords:** Salvage brachytherapy, Prostate cancer, High-dose-rate-brachytherapy, Low-dose-rate-brachytherapy, Androgen deprivation therapy

## Abstract

**Purpose:**

To evaluate efficacy and toxicity after salvage brachytherapy (BT) in prostate local recurrence after radiation therapy.

**Methods and materials:**

Between 1993 and 2007, we retrospectively analyzed 56 consecutively patients (pts) undergoing salvage brachytherapy. After local biopsy-proven recurrence, pts received 145 Gy LDR-BT (37 pts, 66%) or HDR-BT (19 pts, 34%) in different dose levels according to biological equivalent doses (BED_2 Gy_). By the time of salvage BT, only 15 pts (27%) received ADT. Univariate and multivariate analyses were performed to identify predictors of biochemical control and toxicities. Acute and late genitourinary (GU) and gastrointestinal (GI) toxicities were graded using Common Terminology Criteria for Adverse Events (CTCv3.0).

**Results:**

Median follow-up after salvage BT was 48 months. The 5-year FFbF was 77%. HDR and LDR late grade 3 GU toxicities were observed in 21% and 24%. Late grade 3 GI toxicities were observed in 2% (HDR) and 2.7% (LDR). On univariate analysis, pre-salvage prostate-specific antigen (PSA) > 10 ng/ml (p = 0.004), interval to relapse after initial treatment < 24 months (p = 0.004) and salvage HDR-BT doses BED_2 Gy_ level < 227 Gy (p = 0.012) were significant in predicting biochemical failure. On Cox multivariate analysis, pre-salvage PSA, and time to relapse were significant in predicting biochemical failure.

HDR-BT BED_2 Gy_ (α/β 1.5 Gy) levels ≥ 227 (p = 0.013), and ADT (p = 0.049) were significant in predicting grade ≥ 2 urinary toxicity.

**Conclusions:**

Prostate BT is an effective salvage modality in some selected prostate local recurrence patients after radiation therapy. Even, we provide some potential predictors of biochemical control and toxicity for prostate salvage BT, further investigation is recommended.

## Introduction

Patients with intermediate or high risk localized prostate cancer treated with standard dose radiation therapy with or without ADT have a 10 year-rate of biochemical failure (BF) that ranges from 30% to 70%. Some of these patients will die of prostate cancer [1].

Surgery, cryotherapy and brachytherapy are among the most frequently used local salvage treatment options with different degrees of success [2-11]. At the present time, there is no consensus about what the optimal salvage treatment should be in patients who are presumed to have exclusive local recurrence after radiation therapy.

BT as a salvage treatment modality seems to have a potential benefit in patients with local relapse after radiotherapy in other solid tumors [12].

The role of ADT in combination with salvage BT in prostate local recurrence is uncertain because the different studies available comprise a limited and inhomogeneous patient sample [5,6,8-11,13].

Herein, we present the outcome and toxicity in a cohort of patients with locally recurrent prostate cancer treated with salvage BT. Potential predictors of biochemical failure and toxicity after salvage BT were also identified.

## Materials and methods

From January 1993 to July 2007, 56 consecutively patients with biochemical failure by ASTRO or Phoenix definitions [14,15], and local biopsy-proven recurrence after radiation therapy were included in this pooled-analysis study. Eligibility criteria included a negative evaluation of systemic disease, a good urinary and bowel function and a life expectancy more than 5 years.

All information from these patients was collected via retrospective chart review. The prostate biopsy specimen was review by a pathologist of each institution who was familiar with radiation effects on the prostate to avoid false-positive results.

Forty-six patients (82%) were primarily treated with (EBRT) to a median dose of 72 Gy (range, 64 Gy – 78 Gy) and 10 patients (18%) were initially treated with LDR-BT. LDR-patients had received pre-planned D_100_ doses of at least 145 Gy. There was insufficient information of the postimplant D_90_ values.

Other salvage options such as cryotherapy or radical prostatectomy were discussed as well but were refused by the patients. Written informed consent was obtained from the patients for participation in this retrospective review, which was approved by the Institutional Review Boards of the participating institutions.

### Salvage treatment

Patients were excluded for salvage brachytherapy if they did not meet all of the following criteria: Qmax > 15 ml/seg, and a prostate volume < 50 cm^3^.

A total of 19 (34%) patients were treated with salvage ^192^Ir HDR-BT and 37 (66%) patients with salvage ^125^I LDR-BT.

The technique for both permanent and temporal implant followed the local treatment policy of each centre. The characteristics of the patients are shown in Table 1.

**Table 1:** Initial and salvage patient characteristics

**Characteristics**	**At initial presentation**	**At salvage**	
Age (years)	Mean 60	Mean 65	
	Range 50 - 77	Range 60 - 80	
KPS	Mean 80	Mean 80	
	Range 70 - 90	Range 70 - 90	
PSA ng/ml	Mean 17.3	Mean 5	
	Median 10.7	Median 3.7	
	Range 4 - 121	Range 1.1 - 30	
PSA ng/ml	Patients (%)	Patients (%)	
< 10 ng/ml	26 (46%)	51 (91%)	
10-20 ng/ml	18 (32%)	4 (7%)	
> 20 ng/ml	12 (22%)	1 (2%)	
Gleason score	Patients (%)	Patients (%)	
≤ 3 + 3	37 (66%)	9 (16%)	
3 + 4/4 + 3	16 (29%)	14 (25%)	
≥ 4 + 4	3 (5%)	8 (14%)	
Unavailable		25 (45%)	
T stage	Patients (%)	Patients (%)	
T1c	23 (41%)		
T2	26 (46%)		
T3	7 (13%)		
Unknown			
Recurrent		56 (100%)	
D’Amico risk group	Patients (%)	Patients (%)	
Low	24 (43%)		
Intermediate	16 (28.5%)		
High	16 (28.5%)		
Unknown			
Radiation treatment	Patients (%)	Patients (%)	
EBRT	46 (82.%)		
< 72 Gy	24 (43%)		
> 72 Gy	22 (39%)		
LDR-BQT	10 (18%)	37 (66%)	
HDR-BQT		19 (34%)	
Time to biochemical relapse		Patients	Patients
(ASTRO/Phoenix definition)		ASTRO	PHOENIX
< 24 months		6 (11%)	4 (7%)
> 24 months		50 (89%)	52 (93%)
Androgen deprivation therapy	Patients (%)	Patients (%)	
Yes	26 (46%)	15 (27%)	
No	30 (54%)	41 (73%)	
Time to nadir post-RT/BQT			
≤ 6 months	15 (26.8%)		
6 – 12 months	7 (12.5%)		
> 12 months	34 (60.7%)		
Nadir PSA post-radiation	Mean 0.6 ng/ml	Mean 0,5 ng/ml	
	Median 0.5 ng/ml	Median 0.16 ng/ml	
	Range 0.001 – 3 ng/ml	Range 0.001 – 2.9 ng/ml	

*LDR-BQT* = low dose rate brachytherapy; *HDR-BQT* = high dose rate brachytherapy.

The total dose to be administered with salvage brachytherapy, either ^125^I LDR-BT or ^192^Ir HDR-BT is yet to be determined. We use the linear quadratic formulation for dose equivalence. Assuming that the α/β ratio for prostate is about 1.5 Gy [16], the rationale of this study was to use an HDR-BT regimen that provided a BED_2_ of at least 50 Gy EQD2 [17]. Table 2 shows the different HDR dose levels with the corresponding BEDs values using a α/β ratio of 1.5 and 3.0. Patients salvaged with LDR-BT were treated with the same dose recommended by the guidelines for primary tumor in the same setting than other authors have reported.

**Table 2:** Characteristics of salvage BT-HDR dose levels and total BED

** HDR-BT doses**	**BED (α/β = 1.5)**	**BED (α/β = 3)**	**Total BED**_**2 Gy**_	
			**α/β = 1.5 Gy**	**3 Gy**
8.5 Gy × 2	125	65	52	39
6 Gy × 4	132	72	55	43
9 Gy × 2	140	72	59	43
9.5 Gy × 2	154	79	65	48
6 Gy × 5	165	90	69	54
8.5 Gy × 4	227	130	95	78
9 Gy × 4	279	144	116	86
10.5 Gy × 3	280	140	117	84
9.5 Gy × 4	308	158	119	95
13 Gy × 3	419	208	240	206

*HDR-BT* = high dose rate brachytherapy; *BED* = biological equivalent doses;

*α/β* = alfa/beta; *BED*_*2 Gy* =_ biological equivalent doses at 2 Gy per fraction.

Although there is no specific guideline for reirradiation volumes, we followed the GEC-ESTRO recommendations for volume definition and dose prescription [18]. The CTV for HDR-BT included the prostate and seminal vesicles (if appropriate). The CTV for LDR-BT was the prostate only. The following parameters were recommended for all investigators: V100 ≥ 95% of the CTV; D90 > 100% of the prescribed dose. Post-implantation CT dosimetry were recommended to be performed 4 to 6 weeks after BT for those patients treated with LDR-BT according to the recommendations of the AAPM [19].

The mean prescription dose for salvage ^125^I LDR-BT was 145 Gy (range, 120 – 160 Gy). The mean prescription dose for ^192^Ir HDR-BT was 50.5 Gy (range, 17 – 39 Gy), with a mean dose per fraction of 6.2 Gy (median, 5.25 Gy per fraction) in 1–3 implants (range, 1–4 fractions). The mean number of plastic tubes used per implant was 13 (range, 9 – 18).

### Androgen deprivation therapy

At the time of recurrence, neoadjuvant and adjuvant ADT was used in 15 patients (HDR-BT 9 pts; LDR-BT 6 pts) during 3 and 36 months. Only three patients received 36 months hormonal therapy. The follow-up of these three patients from the end of hormonal treatment was 24, 25 and 36 months, respectively.

### Statistical analysis

Cancer control outcomes were calculated using the actuarial method of Kaplan and Meier [20]. BF was defined following the ASTRO or the Phoenix definition [14,15]. Outcomes measured include FFbF and disease-free survival (DFS). DFS was measured from the date of BT salvage until the occurrence of one of the following events, whichever occurred first: BF, local or distant disease, start of any treatment for prostate cancer or any cause of death. Other outcomes evaluated included DMFS, CSS and OS.

#### Univariate and multivariate analysis

For this cohort of patients, univariate and multivariate were performed to identify potential predictors of biochemical control, survival and toxicity following salvage BT. The association of the different variables analyzed with BF and toxicity (initial age, tumor, PSA, and Gleason score, D’Amico risk group at initial treatment [21], initial EBRT dose, nadir PSA post-initial treatment, time to nadir PSA post-initial treatment, interval to relapse after initial treatment, ADT and PSA at salvage, salvage BT dose, and post-salvage PSA nadir) was evaluated with the Chi-square test and their association with outcome measures was determined with the log-rank test and the Cox regression method. Two-sided p values of less than 0.05 were considered statistically significant.

### Follow-up

Patients were followed-up by the Radiation Oncology staff of the participating Institutions every 6 months, for the first 4 years, and on a yearly basis thereafter. Toxicities were recorded and graded using CTCv3.0 [22].

## Results

### Outcomes

**Figure 1:**
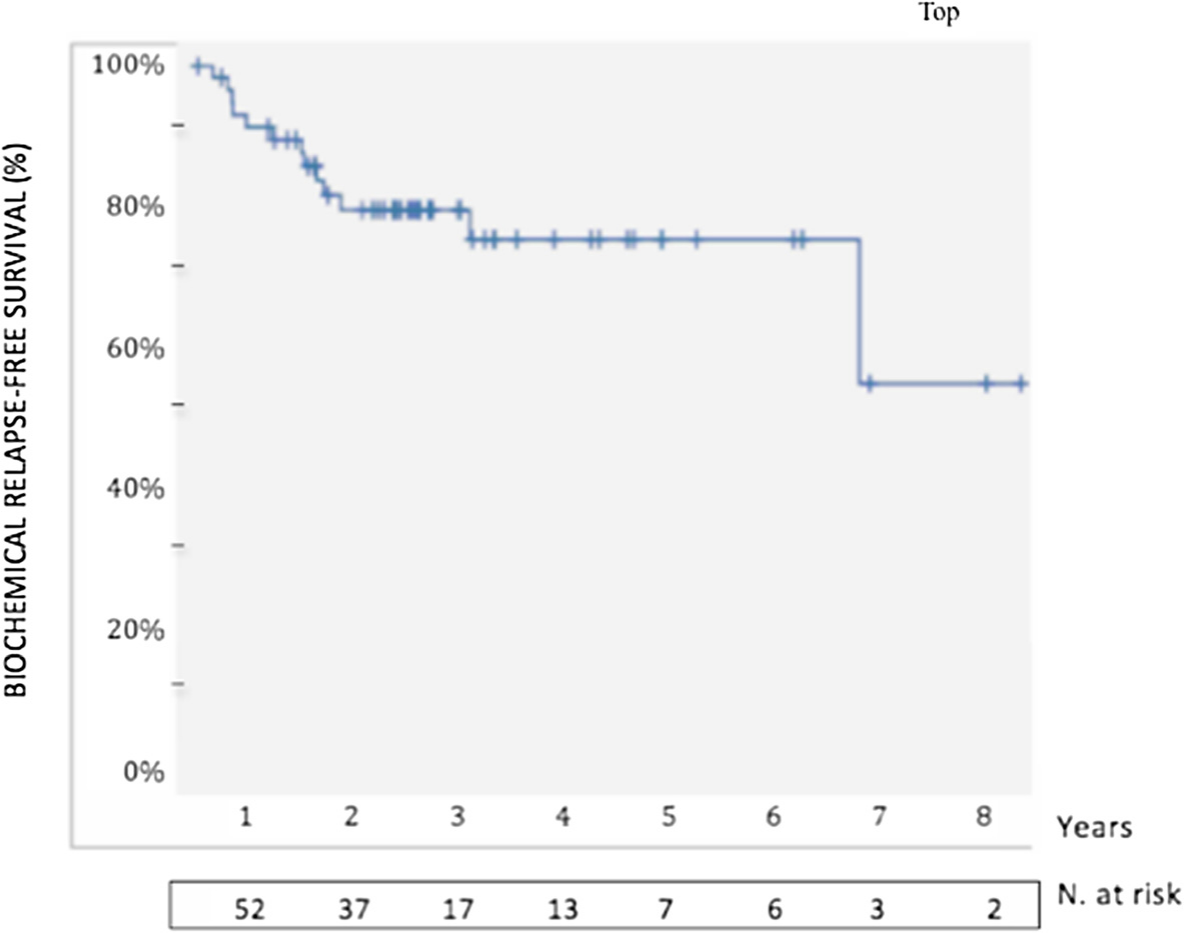
Kaplan-Meier freedom from biochemical failure post-salvage brachytherapy with 5-year estimates.

With a median follow-up of 48 months (range, 25 – 109), the 5-year actuarial FFbF was 77% (Figure 1). The 5-year OS rate was 70%.

Insufficient data were available to assess the IPSS before and after treatment.

On univariate analysis, the factors associated with biochemical failure were psa at salvage > 10 ng/ml (p = 0.004, OR 16.4, 95% CI 1.82-147.7), interval to relapse after initial treatment < 24 months (p = 0.004, OR 16.4, 95% CI 1.82-147.7), and salvage HDR-BT doses BED_2 Gy_ level ≤ 227 Gy (p = 0.012, OR 18, 95% CI 1.75-184.8). Nadir PSA > 1 ng/ml at relapse was of borderline significance in predicting BF (p = 0.087, OR 4.35; 95% CI, 0.73-25.6).

On Cox multivariate analysis, PSA at relapse > 10 ng/ml (p = 0.043, OR 0.181, 95% CI 0.03-0.84), and time to relapse < 24 months (p = 0.031, OR 0.160, 95% CI 0.057-0.571) was strongly associated with biochemical failure.

The factors significantly associated with Grade ≥ 2 urinary toxicity were androgen deprivation therapy at relapse, and HDR-BT at salvage (BED_2 Gy_ level ≥ 227) (Table 3). No factor predictive of rectal toxicity was found.

**Table 3:** Multivariate analysis of factors that may influence Grade ≥ 2 urinary toxicity

**Factor**	**OR (95% CI)**	**p value**
ADT at relapse (yes vs. not)	0.494 (0.245-0.997)	0.049
HDR-BT at salvage	0.459 (0.242-0.870)	0.017
BED_2 Gy_ level ≥ 227 vs		
BED_2 Gy_ level < 227		

*ADT* = androgen deprivation therapy; *OR* = odd ratio; *HDR-BT* = high dose rate brachytherapy; *BED*_2 Gy_ = biological equivalent doses.

At the time of this analysis, only one patient had died of prostate cancer at 25 months from salvage BT.

### Toxicity

#### HDR-BT urinary toxicity

Three out of the 19 patients did not develop any urinary toxicity. Four patients (21%) had Grade 3 toxicity requiring medical support or intervention for symptom relief (urethral stricture that interfered in daily activities requiring periodical dilations n = 4). There were no cases of Grade 4 or greater urinary toxicity.

#### LDR-BT urinary toxicity

Eight out of 37 patients did not develop any urinary toxicity. Nine patients (24%) had Grade 3 toxicity (bladder sphincter spasms requiring narcotics, n = 3; obstructive uropathy requiring TURP after 36 months from salvage therapy, n = 5). There were no cases of Grade 4 or greater urinary toxicity.

#### HDR-BT rectal toxicity

Thirteen of 19 patients did not develop rectal toxicity. One patient (2%) developed a Grade 3 rectal event consisting of persistent rectal bleeding requiring argon laser therapy with complete resolution.

#### LDR-BT rectal toxicity

Nineteen out of 37 patients did not develop any rectal toxicity. One patient (2.7%) had Grade 4 rectal toxicity that consisted of a prostatorectal fistula. This patient required colostomy 24 months after salvage therapy. There were no cases of Grade 3 or 5 rectal toxicities.

The information available to assess the rates of erectile dysfunction after salvage BT was not complete enough to report on this item.

## Discussion

The present study represents, to the best of our knowledge, the largest series of salvage BT ever published. Salvage BT resulted in a 5-year actuarial FFbF of 77% with a corresponding 5-year OS and a CSS rate of 70% and 97%, respectively. These results are comparable with other prior reports on salvage BT (5,6,8-11).

The evidence published at literature of salvage BT varies according to patient and disease-related characteristics and suggests a wide range of outcomes and toxicities. For properly selected patients with local failure after definitive RT, salvage BT can provide long-term disease control comparable with other salvage modalities.

Even the mid-term follow-up of this study is limited, our study of salvage BT found a high 5-year biochemical control rate and it provides durable disease control in a substantial percentage of patients after local recurrence of prostate cancer despite previous definitive RT.

Biochemical failure after primary radiation in localized prostate cancer occurs after 60 months, and therefore a longer follow-up is desirable to analyze how the impact of treatment should be. However, in a salvage treatment (surgery, cryotherapy, brachytherapy), the oncology situation is different probably because of tumor cell becomes more resistant to salvage treatment and therefore many of BF is expected to be in the first 5-year after treatment. This situation is frequently to be observed in other solid tumors.

Chen [9], in a salvage HDR-BT study of 52 patients had a BF of 49% at 5-year. Most of these failures occur before 48 months after treatment. A study with 37 patients from Mount Sinai Medical Center [11] examined the role of salvage LDRB for local failure after prostate radiation therapy. The 5-year BF reported was 35.5%. Only a 11% more of BF was observed at 10-year.

In addition, the mean of follow-up of most relevant salvage brachytherapy series published from 1999 is 52 months (30 – 86 months). The majority of BF of the series are reported at 5-year [3,4,6,8,9], except one author [11], at 10-year (Table 4).

**Table 4:** Salvage brachytherapy series after local failure of radiation therapy for prostate cancer

**Study, year**	**Salvage brachytherapy doses**	**N**	**Pre-salvage PSA (ng/ml)**	**Median f/u (mo)**	**Outcome**	**Grade 3/4 toxicity**
Grado et al., 1999 [4]	LDR 145 Gy	49	5.6	64	34% 5-y bDFS^a^	16%
					56% 5-y bDFS^b^	
Beyer et al., 1999 [3]	LDR 120 Gy ^125^I	17	2.2	62	53% 5-y FFSR^c^	NR
	90 Gy ^103^P					
Wong et al., 2006 [5]	LDR 126 Gy	17	4.7	44	75% 4-y FfbF^c^	47%
Allen et al., 2007 [6]	LDR 97 Gy^d^	12	3.8	45	63% 5-y FfbF^c^	0%
Chen et al., 2013 [9]	HDR 36 Gy	52	9.3	59.6	51% 5-y FfbF^e^	2%
Nguyen et al., 2007 [7]	LDR 137 Gy^f^	25	9.46	46	70% 4-y FfbF^e^	30%
Lee et al., 2008 [8]	LDR 90 Gy^g^	21	3.8	36	38% 5-y FfbF^c^	0%
Aaronson et al., 2009 [10]	LDR 144 Gy	24	3.4	30	89% 3-y FfbF^e^	4%
Burri et al., 2010 [11]	LDR 135 Gy ^125^I	37	5.6	86	65% 5-y FFbF	11%
	110 Gy ^103^P				54% 10-y FfbF^e^	
Current series, 2013	HDR (BED_2 Gy_) 125–419 Gy	56	5	48	77% 5-y FFbF^e^	*HDR GU/GI*
						*G3.......*21%/2%
	LDR 145 Gy					*LDR GU*/*GI*
						*G3.....*24%/0%
						*G4.....*0%/ 2.7%

*LDR* = low dose rate; *HDR* = high dose rate; *bDFS* = biochemical disease-free survival; *FFSR* = freedom from second relapse; *NR* = not reported; *GU* = genitourinary; *GI* = gastrointestinal; *G3* = grade 3; *G4* = grade 4.

^a^Failure defined as two PSA rises above nadir.

^b^Nadir psa < 0.5 ng/ml.

^c^Failure defined as ASTRO definition.

^d^Median dose.

^e^Failure defined as Phoenix definition.

^f^Median minimum peripheral dose.

^g^Minimum peripheral dose.

Our-study revealed that 23% of BF occur in the first 5-year, and hormonal treatment at relapse did not have any influence on BF (p = 0.126, OR 0.298, 95CI 0.063 – 1.4).

At this moment, if there is a suspicious of failure by a rising of PSA after radiation therapy, physical exam with rectal examination, magnetic resonance imaging, bone CT-scan and biopsy-proven histology are strongly recommended before a patient could be offered a salvage treatment. Even, there is an estimation of 70% of relapse are localized at prostate, these biological, clinical and radiological methods are insufficient to demonstrate whether the relapse is local or not. However, in the last years, some prognostic factors have been identified at diagnosis, at initial treatment and at relapse which are associated with lower rates of PSA recurrence by 5 years after any of the salvage local therapies [1]. These factors should be taken into account because they may help to identify the best candidate to offer salvage brachytherapy, as it is suggest by other authors [1].

The consensus of ASTRO (1997) and Phoenix (2005) clearly defined PSA failure after radiation therapy [14,15]. Actually, it is not possible to make definitive statements of PSA failure after salvage brachytherapy because of wide variety of PSA definition reported at literature [3-11]. In our study, the Phoenix criteria were used for relapse after salvage brachytherapy as it was considered by some recent publications [7,9-11].

A number of predictive factors have been related with the risk of subsequent failure and toxicity in patients with local failure treated for salvage [1,9,11,23-26]. To facilitate a better patient selection for salvage BT, our study revealed that the disease-free interval after initial definitive RT, the prior salvage PSA, and salvage HDR-BT doses (BED_2 Gy_ level ≤ 227) were each of significance in predicting biochemical control after salvage BT. Also, nadir PSA > 1 ng/ml after salvage BT was of borderline significance (p = 0.087).

A larger interval of disease-free after initial definitive radiation therapy may suggest local rather than regional/or distant disease recurrence. Chen [9] found a borderline significance in predicting biochemical failure in those patients with a prolonged disease-free interval after initial definitive radiation therapy.

Presalvage PSA is an important predictor of outcome for salvage brachytherapy.

Burri [11] showed a trend for increased freedom from BF in patients with low PSA at the time of salvage LDR. In the postoperative RT literature, earlier salvage external beam RT, results in higher progression-free survival when the presalvage PSA is lower [24]. Similar to other modalities, salvage RP is more effective when the preoperative PSA is lower [23,27].

Although elevated Gleason score [23], extended interval from first recurrence to time to earlier salvage RT [24] and percent positive cores biopsies [9,26] have been found to be a predictor of BF and disease progression, in our salvage BT study we did not find any correlation.

However, only a prospective randomized trial can make definitive statements in PSA failure and prognostic factors after salvage brachytherapy.

The occurrence of relevant treatment adverse effects combined with otherwise good results in terms of cancer control have led to a growing interest in evaluating the impact of treatment on quality of life (QoL).

Surgery, radiation therapy or brachytherapy alone or associated to hormonal treatment is an effective treatment but caused urinary, bowel, or sexual dysfunction with different frequency, duration, and severity. Radical prostatectomy caused urinary incontinence and sexual dysfunction but improved preexisting urinary irritative-obstructive symptoms. External radiotherapy and brachytherapy caused urinary irritative-obstructive adverse effects and some sexual dysfunction. External radiotherapy also caused bowel adverse effects [28,29].

Although salvage treatment (surgery, cryotherapy, brachytherapy) is associated with reasonable cancer control outcomes, sexual, rectal and urinary dysfunction is the mainstay limitations for preserving quality of life (QoL). Actually, there is not prospectively data available in QoL in patients treated with salvage treatment. Most of data reported are based on functional outcomes instead of validated QoL instruments [10,11].

In our study, there were some exclusion criteria based on Qmax and prostate volume, and also, some technical recommendations and a quality parameters of brachytherapy treatment based on GEC-ESTRO guidelines for volume definition and dose prescription. Although these recommendations are insufficient, they are useful to minimize toxicity in urethra, rectal, bladder and sexual function from brachytherapy treatment. While we were unable to document the IPPS on follow-up, at baseline these men had generally good urinary function before BT. The lack of patient questionnaires make this observation circumspect.

Nowadays, there is a strong recommendation to use QoL instruments in future trials of salvage brachytherapy.

Urinary toxicity profile is very important in patients who undergone salvage brachytherapy.

Recently, Chen [9], reported in 52 patients with local recurrence after radiation therapy treated with salvage HDR-BT an acute and late grade 3 GU toxicities in only 2% and 2%, respectively. In this study, all patients received the same salvage treatment with the same total HDT-BT dose and fractionation. The authors did not reported any Grade 4 or 5 GU toxicity. The explanation of this low GU toxicity may be a selection of patients with low urinary toxicity at baseline (even the authors did not reported any based-QoL instruments such as IPSS), and the homogeneous procedure applied in technique, dose and fractionation with HDR-BT. In addition, the urethral-sparing technique included [30] may help limit the rate of urethral stricture. This is the lowest urinary toxicity published with this technique when it is compare with other salvage brachytherapy series [1,4,5,7,10,11,25].

In our study, late grade 3 GU toxicity was observed in 21% and 24% in patients salvaged either with HDR or LDR, respectively. Several factors may explain this GU toxicity, such as, different techniques of brachytherapy, doses and fractionations. Also, we did not quantified any qualified urinary instruments (IPSS) that helped to select a patient with a good urinary function before salvage BT. All these factors may contribute to have a high urinary toxicity.

Even, the GU toxicity in our study are high, the observed rate averaged those described in former reports [1,4,5,7,10,11,25]. The mean average urinary toxicity from salvage BT series are incontinence in 6% and urethral stricture in 17%, respectively. In addition, when we compared with other salvage modalities, the urinary toxicity profile of our study is acceptable. For example, salvage prostatectomy has an associated incontinence rate of 45% (range:0%-80%) and salvage cryoablation can result in incontinence rates ranging from 4.7% to 95% (Table 5).

**Table 5:** Most relevant average toxicities of different salvage techniques

**Salvage technique**	**Incontinence**	**Rectal injury**	**Bladder neck stricture**	**Fistula**
Prostatectomy [23,27]	41%	4.7%	24%	
Cryotherapy [26,31,32]	36%		17%	2.6%
Brachytherapy [1,3-5,7-11]	6%	5.6%	17%	3.4%

A better selection of presalvage BT patients with good urinary function (IPSS < 8 or AUA < 10 scores), and the use of an urethral-sparing technique [30] may help to decrease urinary morbidity.

The results of the present study are far from conclusive due to the wide variability of patients, treatment characteristics regarding BT type, doses and ADT schedule. The retrospective nature of the study and it’s a potential for selection bias may preclude the identification of some patient and treatment-related factors that might influence on outcomes or toxicities.

Nonetheless, even these limitations, we still demonstrated a consistent disease control in a majority of patients. In addition, we identified some potential predicting factors for local control and urinary toxicity that are in line with predictors identified for other salvage modalities. However, we must be cautious to further investigate these predicting factors of outcome and toxicity in prospective trials.

Prospective multi-institutional trials (Radiation Therapy Oncology Group 0526) and the Spanish phase II trial (Anabraq) of salvage BT with or without ADT are currently open to accrual to address these concerns.

## Conclusions

This pooled-analysis study has demonstrated that BT, with careful selection criteria is a feasible salvage treatment after EBRT failure. Even, we identified some predictors of biochemical control and urinary toxicity that may help patient selection for prostate salvage BT, further investigation is recommended in future trials.

## Abbreviations

BT: Brachytherapy; pts: Patients; HDR-BT: High-dose-rate brachytherapy; LDR-BT: Low-dose-rate brachytherapy; BED2 Gy: Biological equivalent doses; ADT: Androgen deprivation therapy; FFbF: Freedom from biochemical failure; OS: Overall survival; GU: Genitourinary; GI: Gastrointestinal; CTCv3.0: Common Terminology Criteria for Adverse Events; α/β: alpha/beta; CTV: Clinical target volume; DFS: Disease-free survival; DMFS: Distant metastasis free survival; CSS: Cause-specific survival; PSA: Prostate antigen specific; Gy: Gray; EBRT: External beam radiotherapy.

## Competing interest

The authors declare that they have no competing interest.

## Authors’ contributions

IH, AP, GS, BG designed the study and the analysis. JP, CG, RMM collected de clinical and dosimetric data. AT, OA, AH contributed to interpretation of data. All authors have given final approval of the manuscript.

## Authors’ information

Presented in part at the ESTRO Congress 2010, Barcelona, Spain.
